# Research Trends in Exercise Interventions for Functional Health in Older Adults: A Bibliometric Analysis (2000–2025)

**DOI:** 10.3390/healthcare14193236

**Published:** 2026-10-01

**Authors:** Yong Zhang, Xiaofang Ying, Zhaofeng Meng, Yajun Zhang

**Affiliations:** 1 School of Medicine, Shaoxing University, Shaoxing 312000, China; zhangyong@usx.edu.cn; 2 School of Business, Shaoxing University, Shaoxing 312000, China; yingxiaofang@usx.edu.cn; 3 College of Physical Education and Health Sciences, Zhejiang Normal University, Jinhua 321004, China

**Keywords:** functional health, exercise interventions, older adults, physical function, physical activity

## Abstract

**Background/Objectives**: Although physical activity and exercise are important for improving functional decline in older adults, the research focuses and trends in this field remain unclear. This study aimed to identify research trends and characteristics in this field and to provide guidance for research and practice. **Methods**: A bibliometric analysis was conducted using data retrieved from Web of Science. A predefined search strategy was applied to identify relevant publications between 2000 and 2025. Data were analyzed and visualized using CiteSpace and VOSviewer. **Results**: A total of 5705 publications were included for the bibliometric analysis. The number of papers published in this field had increased over the past 26 years, with an average of over 400 papers published annually since 2021. The United States had the most published papers, at 2113. China was second, with 941. The focuses of keywords shifted from “disability” prior to 2016 to “mobility”, “quality of life”, “balance”, “walking”, “activities of daily living”, “muscle strength”, “falls” and “executive function” between 2017 and 2019, and finally to “cognitive impairment”, “sarcopenia” and “frailty” since 2020. The keywords with the citation bursts from 2000 to 2010 were “disability”, “risk factors” and “quality of life”; those from 2010 to 2020 were “skeletal muscle”, “community”, “lower-limb function”, “intensity”, “predictors”, “body composition”, “obesity”, “gait”, “blood pressure”, “aerobic exercise”, “balance” and “executive function”; and those after 2020 were “cognitive impairment”, “tai chi”, “management”, “instrumental activity”, “scale”, “muscle mass” and “healthy ageing”. **Conclusions**: Exercise intervention for functional health in older adults remains a hot topic in research. Research institutions and teams in this field are diverse, with the United States and China emerging as the leading countries. Specific aspects of physical function performance related to disability and quality of life—including cognitive and executive functions—and the corresponding exercise interventions are the focuses of current research.

## 1. Introduction

The global population is ageing rapidly. By 2050, there will be 1.6 billion adults aged 65 and over, accounting for around 16% of the population, and 459 million people aged 80 and over [1]. It is well known that multimorbidity and functional decline associated with ageing severely impact the quality of life of older adults and their families [2] and pose significant medical and socioeconomic challenges [3]. While the mechanisms underlying the coexistence of multiple chronic diseases associated with ageing are complex [4], promoting functional health remains a key strategy for improving the quality of life among older adults and alleviating medical and economic pressures [2], and it is the primary pathway to achieving active and healthy aging [5,6].

In fact, since the World Health Organization introduced the ICF classification in 2001, emphasizing the importance of functional health [7], a large number of studies have been conducted on improving and promoting functional health among older adults. These studies primarily focused on the content framework of functional health [8,9,10], assessment methods [9,11], and intervention strategies [5,12]. Among these, physical activity and exercise have received widespread attention as important non-medical interventions [9,13,14]. Studies have found that physical activity and exercise could improve muscle mass and strength [9,15], balance [9], mobility [16,17], gait [18,19], the risk of falls [20], cardiorespiratory function [18,21], frailty [15], cognitive function [22,23], executive functions [24], mental health [25], and activities of daily living [9,26]. The Guidelines for Exercise Interventions to Promote Functional Health in Older Adults also provided a detailed overview of the evidence and methods for exercise interventions targeting functional decline in older adults [27]. However, it is still necessary to analyze the research trends of exercise interventions for the functional health of the elderly, especially the changes in research topics since the ICF was released in 2001, as well as the research activities in different countries and regions. The shifts in research topics not only reflect current research priorities in the field, but also indirectly highlight the pressing practical issues that need to be addressed. Against this backdrop, and considering that the ICF was published in 2001, this study conducted a bibliometric analysis of research on exercise interventions for functional health in older adults since 2000. It examined the trends and characteristics of research in this field, identified key research topics, and provided guidance for future research and practice in exercise interventions for functional health in older adults.

## 2. Materials and Methods

### 2.1. Search Strategy

On 7 January 2026, documents related to exercise interventions for the functional health of the elderly were retrieved from the Web of Science Core Collection (all editions) using a set of predefined topic-based search terms.

The search terms fell into four categories: namely, elderly individuals, exercise, function, and health. In the exercise category, physical activity was also frequently used in health promotion. In fact, exercise and physical activity are not synonymous, but they are related in some ways. Physical activity is defined as any bodily movement produced by skeletal muscles that results in energy expenditure. Physical activity can be categorized into occupational, sports, conditioning, household, or other activities. Exercise is a subset of physical activity that is planned, structured, and repetitive and has as a final or an intermediate objective the improvement or maintenance of physical fitness [28]. Physical fitness is a set of attributes that are either health- or skill-related. Given the current state of exercise and health interventions, the terms “exercise”, “physical activity” and “training” were considered in the exercise category. Ultimately, the specific search terms included “elderly”, “older adults”, “older people”, “older men”, “older women”, “exercise”, “physical activity”, “training”, “function” and “health”. During the retrieval process, the relationship between terms in different categories was “AND”, while the relationship between terms within the same category was “OR”. The search formula was shown in Box 1. The search results were refined using “publications years (2000–2025) “, “document types (article)”, and “language (English)”. Subsequently, the records were screened by reviewing the titles and abstracts. Finally, the records that were included were exported in “plain text file” and “full record and cited references”.

Box 1 **Search formula.**

(TS = (older adults) OR TS = (older people) OR TS = (older men) OR TS = (older women) OR TS = (elderly)) AND (TS = (exercise) OR TS = (physical activity) OR TS = (training)) AND (TS = (function)) AND (TS = (health)).

### 2.2. Inclusion and Exclusion Criteria

The search results were limited to articles published in English between 2000 and 2025. Two reviewers (Yong Zhang and Xiaofang Ying) independently screened the literature records, and differences were resolved by consensus with the third reviewer (Yajun Zhang). Articles that primarily investigated or reported on exercise and functional health in older adults were included. Articles that were non-original studies, including reviews, clinical guidelines or studies with animals as subjects, were excluded. Additionally, studies not involving elderly subjects and research content unrelated to exercise and functional health were also excluded. Finally, the data files of the articles that met the criteria were exported for conducting the bibliometric analysis. The detailed flow chart can be found in Figure 1.

### 2.3. Data Analysis

VOSviewer (v. 1.6.20), CiteSpace (v. 6.3. R1), and Microsoft Office Excel 2019 were employed to analyze the data files of articles meeting the criteria. VOSviewer is bibliometric software developed by van Eck and Waltman at the Centre for Science and Technology Studies at Leiden University in the Netherlands [29]. This Java-based software is freely available and excels at visualizing and analyzing large datasets. CiteSpace, created by Chen et al. [30], serves as a bibliometric analysis and visualization tool capable of exploring collaborative relationships, research priorities, internal structures, core domains, and emerging trends within specific fields. The data were analyzed both quantitatively and qualitatively. The number of articles and their distribution across years, countries, institutions and authors were described. Furthermore, the corpus was analyzed to generate various scientific landscapes and networks. Visual network analyses were conducted for authors, institutions and countries, respectively. A citation burst analysis was also performed by country. The most frequently cited journals and papers were presented. In the trend analysis, clustering and co-occurrence analyses were performed on keywords and terms found in titles and abstracts. The keywords with the strongest citation bursts were also analyzed.

## 3. Results

### 3.1. Annual Scientific Production

A total of 11,066 articles were retrieved from the Web of Science Core Collection by limiting language (English), time (2000–2025), and article type (article). After screening the titles and abstracts, 5361 articles were excluded. Of these, 4964 were irrelevant, 137 were study protocol articles, 107 were non-elderly subjects, 75 were reviews or meta-analyses, 63 were animal studies, 12 were retracted articles, two were duplicates and one was a guideline article. Ultimately, 5705 articles were included in the bibliometric analysis. A simple study flow chart is shown in Figure 1.

To analyze the development trend of studies on functional health and exercise interventions in older adults over the past two decades, we plotted the number of publications by year (Figure 2). Obviously, the number of articles published in this field has risen over the past 26 years. In particular, the average annual number of articles published in this field reached 140 after 2009, more than 300 after 2017, more than 400 after 2021, and peaked at 482 in 2025.

### 3.2. Distribution of Authors, Countries, and Institutions

A total of 26,199 authors from 5914 organizations across 94 countries published articles on functional health and exercise interventions in older adults. The top 20 authors by publication volume averaged 41 papers and 4056 citations per person. Five authors published over 50 related articles (Table S1). Newman AB had the largest number of publications (71, total link strength = 287, citations = 9786), followed by Kritchevsky SB (57, total link strength = 268, citations = 7818), Pahor M (57, total link strength = 286, citations = 7348), Rejeski J (55, total link strength = 195, citations = 5289), and Simonsick EM (51, total link strength = 176, citations = 6967).

From the author network involved in the study of functional health and exercise interventions in older adults (Figure S1), there were a significant number of teams engaged in related research. In the networks generated by CiteSpace analysis, centrality serves as a key parameter for measuring the importance of nodes within the networks. Nodes with centrality values exceeding 0.1 are designated as critical nodes and are regarded as high-centrality nodes. As shown in Table S1, there was little difference in the number of publications among the top 10 most productive researchers. Among the top 20 authors, only two (Newman AB: 0.12 and Liu-Ambrose T: 0.11) had a centrality score greater than 0.1.

In terms of country distribution and collaboration networks, the United States (2113 papers, centrality = 0.33), China (941 papers, centrality = 0.12), the United Kingdom (572 papers, centrality = 0.15), Australia (465 papers, centrality = 0.11), Canada (399 papers, centrality = 0.13), Japan (361 articles, centrality = 0.10), and Spain (321 papers, centrality = 0.18) made significant contributions and exerted considerable influence in this field (Table S2, Figure 3 and Figure S2). Additionally, we used VOSviewer software to perform a clustering analysis of the countries. Each element consists of a circle and a label, with the color indicating the category and time range to which it belongs (Figure S2 and Figure 4). Figure 4 showed three color-coded clusters (purple, green, and yellow) representing the three time periods of 2000–2016, 2018–2020, and 2022–2025, respectively. The purple cluster primarily included the United States, the Netherlands, and Finland. The green cluster mainly comprised Australia, England, and Japan. The yellow cluster consisted primarily of China. Research in this field shifted from being dominated by the USA in 2016 to being dominated by China in 2022. Citation data from the articles included revealed similar findings (Figure S3), indicating that China currently makes significant contributions and exerts considerable influence in this field. Figure 5 also showed that 15 out of 94 countries had the strongest citation bursts, with the United States and China leading the way. From a temporal perspective, research in this field experienced citation bursts in the United States from 2000 to 2012 and in China from 2022 to 2025.

The analysis also revealed that research institutions and collaboration networks in this field were diverse (Table S3 and Figure S4). In terms of publication output, the University of Pittsburgh, Wake Forest University, the University of Illinois, the University of California, Stanford University, and the National Institute on Aging were the leading institutions. The University of Jyväskylä, the University of British Columbia, and the University of Sydney also made significant contributions. It was worth noting that several Chinese institutions appeared in the visualized collaboration network (purple cluster) (Figure S4), such as Peking University, the University of Hong Kong, Shanghai Jiao Tong University, and Shanghai University of Sport. Institutions with centrality greater than 0.10 included the University of Pittsburgh (0.28), the University of Illinois (0.16), the Karolinska Institute (0.14), the University of Melbourne (0.14), and Duke University (0.11) (Table S3).

### 3.3. Influential Journals and Articles

Table S4 lists the top 20 most-cited journals, while Table S5 lists the 20 journals with the most-cited articles. Ten journals appeared in both Table S4 and Table S5 (Table S6). Among them, “Journals of Gerontology Series A-Biological Sciences and Medical Sciences” and “Journal of the American Geriatrics Society” had a high number of citations and a high number of cited articles. In addition, Table S7 lists the 50 most influential articles on functional health and exercise interventions in older adults, which are regarded as classics in this field. Reading these articles is of great significance in enabling a greater understanding of the important research directions in the field of functional health and exercise interventions for older adults.

### 3.4. Co-Occurrence Analysis for Author Keywords

The authors’ keyword co-occurrence network was constructed using VOSviewer software (Figure S5). A total of 7028 keywords were extracted, of which 57 appeared 50 or more times. After excluding terms related to search strategies and study designs, 36 terms remained (Table S8). These co-occurring terms formed four clusters. The first cluster primarily included cognition (327), cognitive function (296), depression (175), dementia (155), activities of daily living (144), executive function (96), mental health (96), cognitive impairment (86), mild cognitive impairment (73), sedentary behavior (67), cognitive decline (60), and Alzheimer’s disease (59). The second cluster primarily included quality of life (273), rehabilitation (142), falls (124), balance (103), walking (83), osteoarthritis (64), strength (60), gait (59), and tai chi (57). The third cluster primarily included sarcopenia (197), muscle strength (131), obesity (119), physical fitness (101), resistance training (97), body composition (85), health-related quality of life (68), and nutrition (53). The fourth cluster primarily included frailty (254), disability (171), mobility (142), physical performance (122), healthy aging (73), mortality (62), and gait speed (59). In addition, we used VOSviewer software to perform a cluster analysis of keywords over time (Figure S6). These keywords gradually shifted from “disability” and “rehabilitation” in 2016 to “mobility”, “balance”, “walking”, “activities of daily living”, “muscle strength”, “falls” and “executive function” in 2017–2019, and then to “sarcopenia”, “frailty”, “cognition” and ”healthy aging” after 2020.

### 3.5. Co-Occurrence Analysis for All Keywords

The VOSviewer software was also used to construct a co-occurrence map of all keywords (Figure S7). A total of 11,575 keywords were extracted, of which 189 appeared ≥50 times. After removing the terms related to the search strategy and study design, 91 terms remained, of which the top 50 were listed in Table S9. Cluster 1 mainly included dementia (608), cognitive function (487) and impairment (392), Cluster 2 mainly included falls (349), balance (322) and walking (224), Cluster 3 mainly included strength (491), sarcopenia (326) and muscle strength (324), Cluster 4 mainly included quality of life (525) and depression (401), and Cluster 5 mainly included disability (847) and mortality (629), mobility (408) and frailty (392). These keywords also underwent a shift from “disability” before 2016 to “mortality”, “quality of life”, “strength”, “balance” and “falls” between 2017 and 2019, and then to “cognitive function”, “sarcopenia” and “frailty” after 2020 (Figure S8).

### 3.6. Co-Occurrence Analysis for Terms in Titles and Abstracts

A co-occurrence term network for titles and abstracts was constructed (Figure 6). A total of 83,768 co-occurrence terms were extracted, of which 712 appeared 50 or more times. Based on relevance scores, the top 60% of the most relevant terms were selected, resulting in 427 terms that formed four clusters (links: 74,169; total link strength: 733,890). After excluding terms related to search strategies and study designs, as well as terms irrelevant to the content, 160 terms remained. Table S10 lists the top 50 terms. Cluster 1 primarily consisted of balance (562), home (497), and pain (463). Cluster 2 primarily consisted of strength (798), body mass index (538), and muscle strength (488). Cluster 3 mainly consisted of disability (725), daily living (597), and depression (496). Cluster 4 mainly consisted of cognitive decline (397), memory (381), and executive function (331), while Cluster 5 mainly consisted of Short Physical Performance Battery (269) and SPPB (189). Similar to the keywords, the trends evolved from “disability”, “body mass index” and “pain” before 2016, to “strength”, “balance”, “daily living”, “home”, “depression” and “muscle strength” between 2017 and 2019, and then to “cognitive decline” and “executive function” after 2020 (Figure 7).

### 3.7. Keywords with the Citation Bursts

The top 30 keywords for citation burst were shown in Figure 8. Between 2000 and 2025, “disability”, “skeletal muscle”, “community”, “lower extremity function” and “risk factors” were the top 5 keywords with the citation bursts. From 2000 to 2010, the focus was on overall physical function, with the citation bursts of keywords including “disability”, “risk factors” and “quality of life.” Since 2010, there had been a greater emphasis on specific physical function outcomes and exercise interventions, with the citation bursts of keywords such as “skeletal muscle”, “community”, “lower extremity function”, “intensity”, “predictors”, “body composition”, “obesity”, “gait”, “blood pressure”, “aerobic exercise”, “balance” and “executive function”. However, since 2020, the keywords with citation bursts had been “cognitive impairment”, “tai chi”, “management”, “instrumental activity”, “scale”, “muscle mass” and “healthy ageing”.

## 4. Discussion

### 4.1. General Information

By analyzing the characteristics of articles published since 2000 on functional health and exercise interventions in older adults, this study aimed to describe the state of research in the field and provide guidance for future research and clinical practice. Overall, between 2000 and 2008, there were relatively few publications in the field of functional health and exercise interventions for older adults, with an average of fewer than 100 articles per year. However, since 2009, the number of research publications in this field had increased sharply, exceeding 300 in 2017. In particular, since 2021, the annual number of publications has surged to over 400, and this growth is expected to continue in the future. From the author network, there were many research teams active in this field. The publication number of the top 10 researchers were relatively close, and among the 20 most productive authors, only two (Newman AB: 0.12, Liu-Ambrose T: 0.11) had a centrality score greater than 0.1.

A visualization analysis by country showed that the United States published the most papers in this field—2.2 times as many as China, which ranked second, and 3.7 times as many as the United Kingdom, which ranked third. However, in terms of centrality, the United States, China, the United Kingdom, Australia, Canada, Japan, and Spain all had centrality values greater than 0.1, indicating that these countries had made significant contributions and exerted considerable influence in this field. Furthermore, based on changes in publication periods and citation periods, research in this field shifted from being dominated by the United States around 2016 to being dominated by China around 2022. Countries with citation bursts also confirmed that the United States was the leading country in research in this field from 2000 to 2012, while China was the leading country from 2022 to 2025. Furthermore, as shown in the research institutions and their network map, the University of Pittsburgh, Wake Forest University, the University of Illinois, the University of California, Stanford University, and the National Institute on Aging in the United States were the leading institutions in this field, while the University of Jyväskylä in Finland, the University of British Columbia in Canada, and the University of Sydney in Australia also made significant contributions. It is also worth noting that several Chinese research institutions appeared in the visualized collaboration network map.

We speculate that the distribution and trends in research in this field are related to the aging process and reflect the focus of the academic community and the government on aging issues. In 2015, the World Health Organization introduced the concept of healthy ageing to address the challenge. According to WHO, healthy ageing is not just the absence of disease, but the process of developing and maintaining the functional ability that enables well-being in older age [31]. The key to healthy ageing is maintaining functional ability despite having chronic conditions. Improvements in functional health during old age can reduce the demand for health care services and lead to healthier older people who can live independently for longer and require less care from family and society. Further, adequate physical functioning even allows them to contribute to their families and society as members of the workforce. As a result, policy makers in any country facing an ageing population would be wise to implement integrated policies to promote healthy ageing. For example, in 2016, the State Council of China officially released the blueprint of Healthy China 2030. This document identified physical activity and exercise as part of a healthy lifestyle and listed them as one of the key indicators of “Healthy China 2030”. In 2019, the State Council of China also issued a corresponding action plan and established specific goals and benchmarks for translating the blueprint into action [32]. Against this backdrop of practical challenges and national policies, the importance of physical activity and exercise as non-medical interventions for functional health received widespread attention, and research in this field developed rapidly, as might be expected.

Overall, the regional characteristics of research in this field were closely linked to the progression of population aging, as evidenced by the contrast between developed countries, such as the United States, and developing countries, such as China. Research institutions and teams were diverse, reflecting both the flourishing state of research in this field and the practical needs it addresses. Additionally, in terms of influential journals, the “Journals of Gerontology Series A-Biological Sciences and Medical Sciences” and the “Journal of the American Geriatrics Society” had high numbers of publications and citations, making them worth paying attention to. The study also listed the 50 most influential articles to help researchers quickly understand the developments in the field.

### 4.2. Hotspots and Frontiers

Keyword analysis can help us understand the hotspots and frontiers in a certain field. The co-occurrence frequency of keywords reflects research hotspots in the field, and changes in the frequency over time reflect the field’s development, helping to analyze the potential causes of shifts in research within the field. This study conducted co-occurrence analyses of both author keywords and all keywords. The co-occurrence network for author keywords formed four clusters. The first cluster was primarily associated with cognitive and mental functioning. The second cluster primarily focused on quality of life and specific functional aspects. The third cluster was primarily related to muscle and body composition, including sarcopenia, muscle strength, obesity, physical fitness, resistance training, body composition, and nutrition. The fourth cluster was primarily related to overall physical function and included frailty, disability, mobility, physical performance, healthy aging, mortality, and gait speed. These keywords gradually shifted from “disability” and “rehabilitation” in 2016 to specific physical functions in 2017–2019, such as “mobility”, “balance”, “walking”, “activities of daily living”, “muscle strength”, “falls”, “executive function”, and then to “sarcopenia”, “frailty” and “cognitive function” since 2020. The results of the co-occurrence network for all keywords were largely consistent with those for the authors’ keywords. Cluster 1 primarily consisted of cognitive function. Cluster 2 primarily consisted of specific physical functions such as falls, balance, and walking. Cluster 3 primarily consisted of muscle function. Cluster 4 primarily consisted of quality of life, and Cluster 5 primarily consisted of disability. It also evolved from “disability” before 2016 to “mortality”, “quality of life”, “strength”, “balance” and “falls” between 2017 and 2019, and then to “cognitive function”, “sarcopenia” and “frailty” since 2020.

Additionally, the study analyzed co-occurrence terms in titles and abstracts. Cluster 1 primarily consisted of “balance”, “home” and “pain”; Cluster 2 primarily consisted of “strength” and “body mass index”; Cluster 3 primarily consisted of “disability”, “daily living” and “depression”; Cluster 4 primarily consisted of “cognitive decline” and “executive function”; and Cluster 5 primarily consisted of “Short Physical Performance Battery (SPPB)”. In terms of time periods, the focus shifted from “disability”, “body mass index” and “pain” prior to 2016 to “strength”, “balance”, “daily living”, “home” and “depression” between 2017 and 2019, and then to “cognitive decline” and “executive function” since 2020.

Keyword burst detection identifies keywords that have experienced a sudden increase in frequency over a short period of time. This reveals changes in research focuses over time and reflects evolving hotspot trends. The keywords with the strongest citation bursts showed a similar trend to the keyword co-occurrence map. Between 2000 and 2010, the focus was primarily on broad concepts such as “disability”, “risk factors” and “quality of life.” After 2010, there was greater emphasis on specific physical function outcomes and practical intervention strategies, with keywords including “skeletal muscle”, “community”, “lower extremity function”, “intensity”, “predictors”, “body composition”, “obesity”, “gait”, “blood pressure”, “aerobic exercise”, “balance” and “executive function.” Since 2020, “cognitive impairment”, “tai chi”, “management”, “instrumental activity”, “scale”, “muscle mass” and “healthy aging” had consistently been in citation bursts. It was worth noting that, from the perspective of exercise intervention methods and intervention concepts, terms such as “aerobic exercise”, “intensity”, “resistance exercise”, “tai chi” and “healthy aging” had appeared in the top 30 keywords with the citation bursts.

Overall, between 2000 and 2025, research keywords in this field primarily covered three areas. First, specific physical functions such as balance, muscle, walking, mobility, and falls; second, cognitive, emotional, and executive functions; and third, overall functional outcomes such as disability, frailty, quality of life, and activities of daily living. Additionally, keywords relating to exercise interventions primarily covered aerobic exercise, resistance training, tai chi and sedentary behavior. These findings suggest that mobility and executive function are essential for older adults to perform daily activities and maintain their quality of life. Specific areas for exercise intervention include muscle strength, balance, walking, fall prevention and cognitive function, all of which are closely related to mobility and executive function. The primary types of exercise for intervention are aerobic exercise, resistance training and tai chi, and intensity is an important variable. In terms of outcome measures, SPPB is a key focus. These keywords and their evolution over this period could be related to shifts in the research focuses and research paradigms in this field.

### 4.3. Shifts in Research Focuses

In terms of research developments in the field of functional health and exercise interventions for older adults, the focuses shifted from examining the positive relationship between exercise and functional health to more targeted, practical interventions. These included research on targeted intervention methods for specific functional impairments, targeted outcome assessment methods, and the feasibility and adherence of intervention programs. These findings can provide direction guidance for scientific research and clinical practice in this field.

Early research focused on the potential role of physical activity and exercise in improving cognitive decline [33,34], mobility limitations [18], disability [18], mortality [18], and survival [18,19] among older adults. Subsequently, the focuses shifted to specific physical functional impairments in older adults and corresponding intervention methods. These specific physical functional impairments primarily included frailty [15,35], sarcopenia [36,37], falls [17,20], mental health disorders [25,38], cognitive decline [22,39,40], and executive function decline [40]. The intervention methods mainly involved aerobic exercise [24,41], resistance training [24,37,42], a combination of aerobic and resistance training [24], multicomponent exercise [43], and structured physical activity [16,23]. Furthermore, exercise intervention modality targeting specific functional impairments were recommended [15,27]. For frailty and sarcopenia, resistance training, balance exercises, gait retraining, and multicomponent exercise were recommended. For falls, resistance training, balance exercises, multicomponent exercise, and Tai Chi exercises were recommended. For cognitive impairment, walking, aerobic exercise, resistance training, and multicomponent training were recommended. For mobility limitations, aerobic exercise, resistance training, balance exercises, gait retraining, and multicomponent exercise were recommended. In addition to refining intervention methods, attention has also been focused on outcome assessment, with particular emphasis on frailty status [44], muscle function [45], physical performance [46,47]. Furthermore, adherence to exercise programs is the final step in the intervention process. Some studies also conducted systematic reviews on older adults’ acceptance of physical activity participation [48,49] and the factors influencing such participation [50]. The results indicated that physical activities that were enjoyable, social, and offered short-term benefits were more readily accepted, while key influencing factors included an individual’s functional capacity, perceived risk of injury from physical activity, the awareness of the health benefits of physical activity, as well as the safety of the neighborhood environment and the accessibility and convenience of exercise facilities. Regarding solutions, the studies also suggested that exercise interventions using behavior change techniques and virtual reality-based techniques were worth encouraging [51,52]. These shifts in research focuses indicate that studies on functional health promotion through exercise for the elderly have moved from being primarily based on evidence research to being more focused on practical application research, especially for the elderly population characterized by multiple functional impairments.

### 4.4. Shifts in Research Paradigms

In this field, the research paradigm evolved from a focus on cross-sectional and cohort studies (correlational research) to evidence-based research centered on RCTs and clinical trials (causal research), and finally to a focus on systematic reviews, meta-analyses, and guidelines and consensus statements. Among these, cross-sectional and cohort studies primarily focused on the relationship between physical activity and cognition [33,34,53,54], the relationship between muscle mass and strength and physical function [55,56,57], and the relationship between physical activity and disability and survival [18,19]. RCTs and clinical trials primarily conducted causal studies on the effects of physical activity [58], resistance training [42], and aerobic training [41] on cognition. Subsequently, in light of the multiple functional declines observed in older adults, attention shifted to the effects of mixed exercise interventions on physical function in this population [16,43]. As research accumulated, systematic reviews and meta-analyses on the effects of physical activity and exercise on sarcopenia [36,37], fall prevention [20,37], cognitive function [39,40], and physical function [9] in older adults have been widely conducted. Guidelines and consensus statements had also emerged regarding physical frailty management [15], fall prevention and management [17], and related exercise intervention programs [27,59] for older adults.

Overall, research in the field of functional health and exercise interventions for older adults has evolved from correlational studies to causal studies, and on to the dissemination of practice guidelines. While research previously focused on theoretical and mechanistic studies, it now places greater emphasis on practical implementation. A recent meta-analysis of randomized controlled trials once again demonstrated the benefits of functional exercise for the physical and psychosocial capabilities of women over the age of 60 [60]. However, further research is needed on how to develop appropriate exercise intervention programs for older adults with multiple functional declines, as well as on adherence to these programs. Although multicomponent exercise is recommended, the design and implementation of tailored exercise programs still require careful consideration. Therefore, based on the ICF framework, we believe it is necessary to develop a content framework system and a corresponding exercise prescription system for exercise interventions aimed at promoting functional health in older adults. These systems should integrate the approaches and physiological mechanisms of exercise interventions targeting functional health. In practice, exercise programs can be tailored to older adults based on their specific physical functioning and social and environmental context, and the implementation of these exercise interventions should also be monitored.

### 4.5. Strengths and Limitations

A bibliometric analysis based on visualization can help scholars quickly identify research focuses and trends. To the best of our knowledge, this is the first bibliometric analysis to focus on exercise interventions for functional health in older adults. This visual analysis of the literature offers researchers insight into the themes, hotspots and trends of functional health and exercise interventions for older adults. However, this study also has several limitations that need to be addressed. Firstly, the study only retrieved literature from the Web of Science Core Citation Database. While this database is one of the most authoritative tools for searching scientific and technical literature, it cannot cover all studies on exercise interventions for functional health in older adults. Secondly, due to limitations in the period over which data was retrieved, some recent publications that had not yet been indexed in the database, or that had not yet accumulated citations, could also introduce potential bias. Thirdly, including only English-language literature could lead to potential language and geographical biases, especially when comparing scientific outputs across different countries.

## 5. Conclusions

Research on exercise interventions for functional health in older adults is expanding rapidly. The United States and China are leading nations in this field, and there is a growing diversity among global research institutions and teams reflecting both the flourishing state of research in this area and the practical need for it. In terms of research topics, the focus in this field has shifted from an early emphasis on “disability” to specific physical functions such as “mobility”, “balance”, “walking”, “activities of daily living”, “muscle strength” and “falls”, and then to “cognitive function”, “executive function”, “sarcopenia” and “frailty” after 2020. Overall, specific aspects of physical function performance related to disability and quality of life—including cognitive and executive functions—and the corresponding exercise intervention are the focuses of current research.

## Figures and Tables

**Figure 1:**
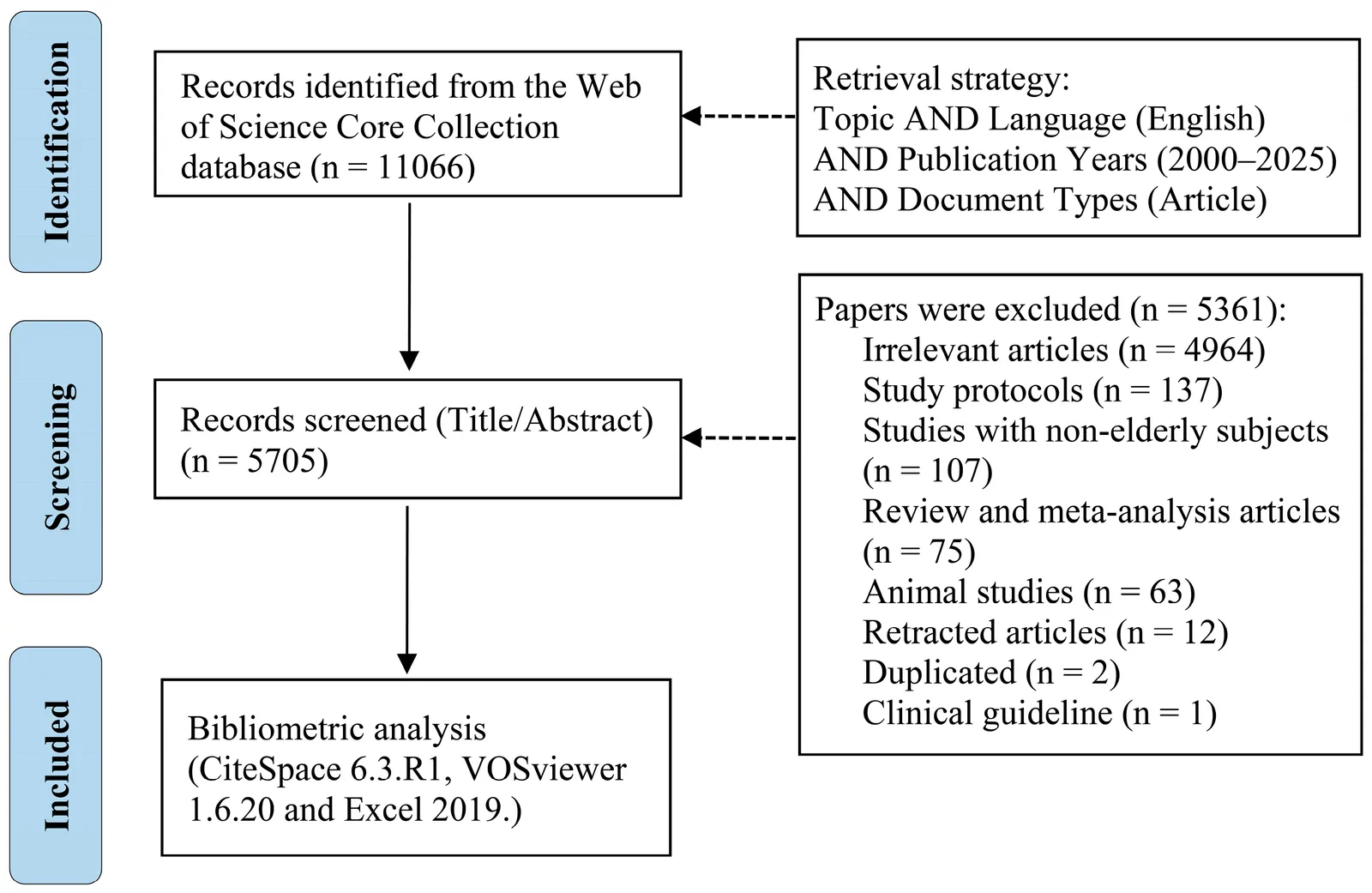
Study flow chart.

**Figure 2:**
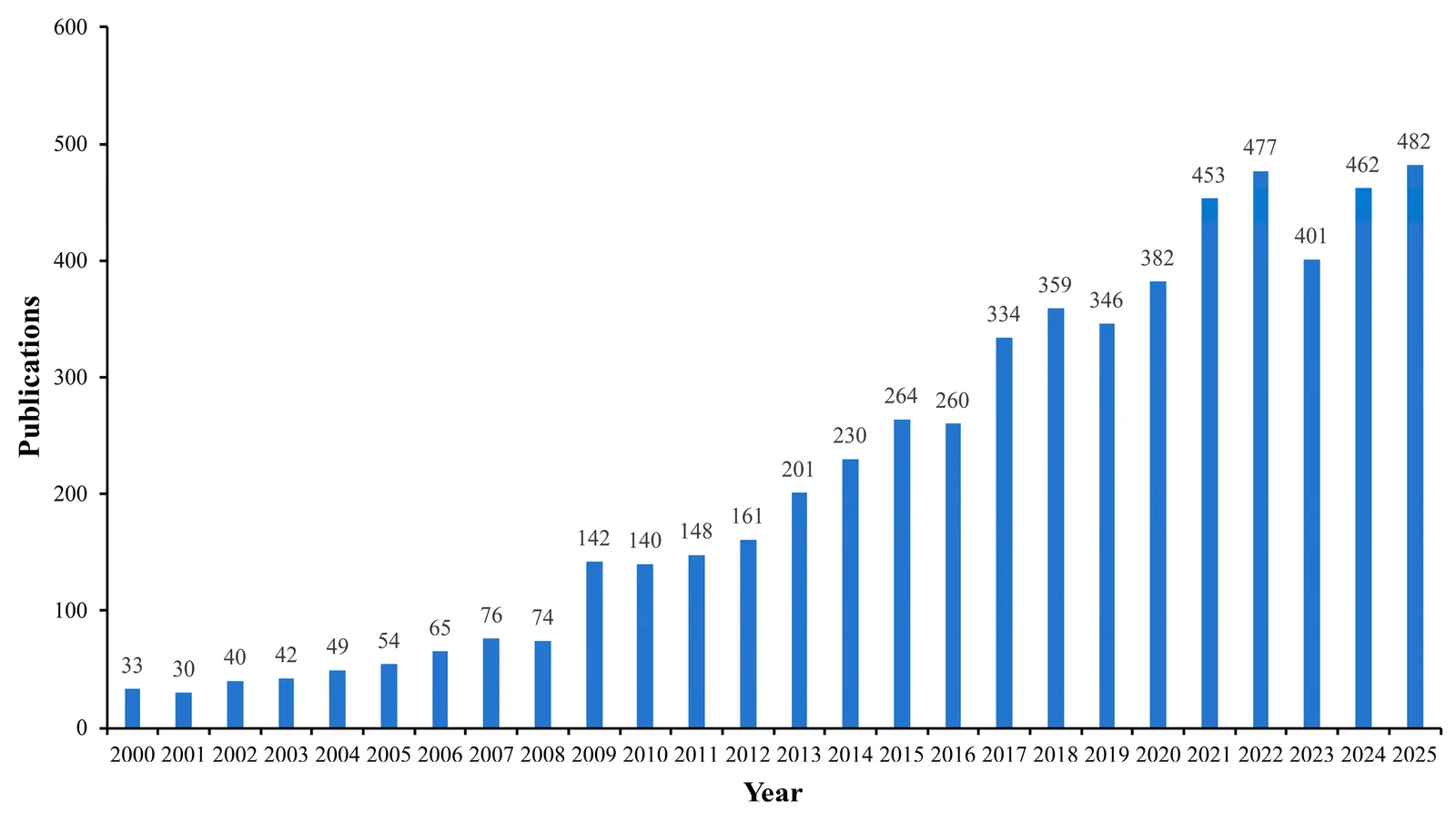
Number of annual publications.

**Figure 3:**
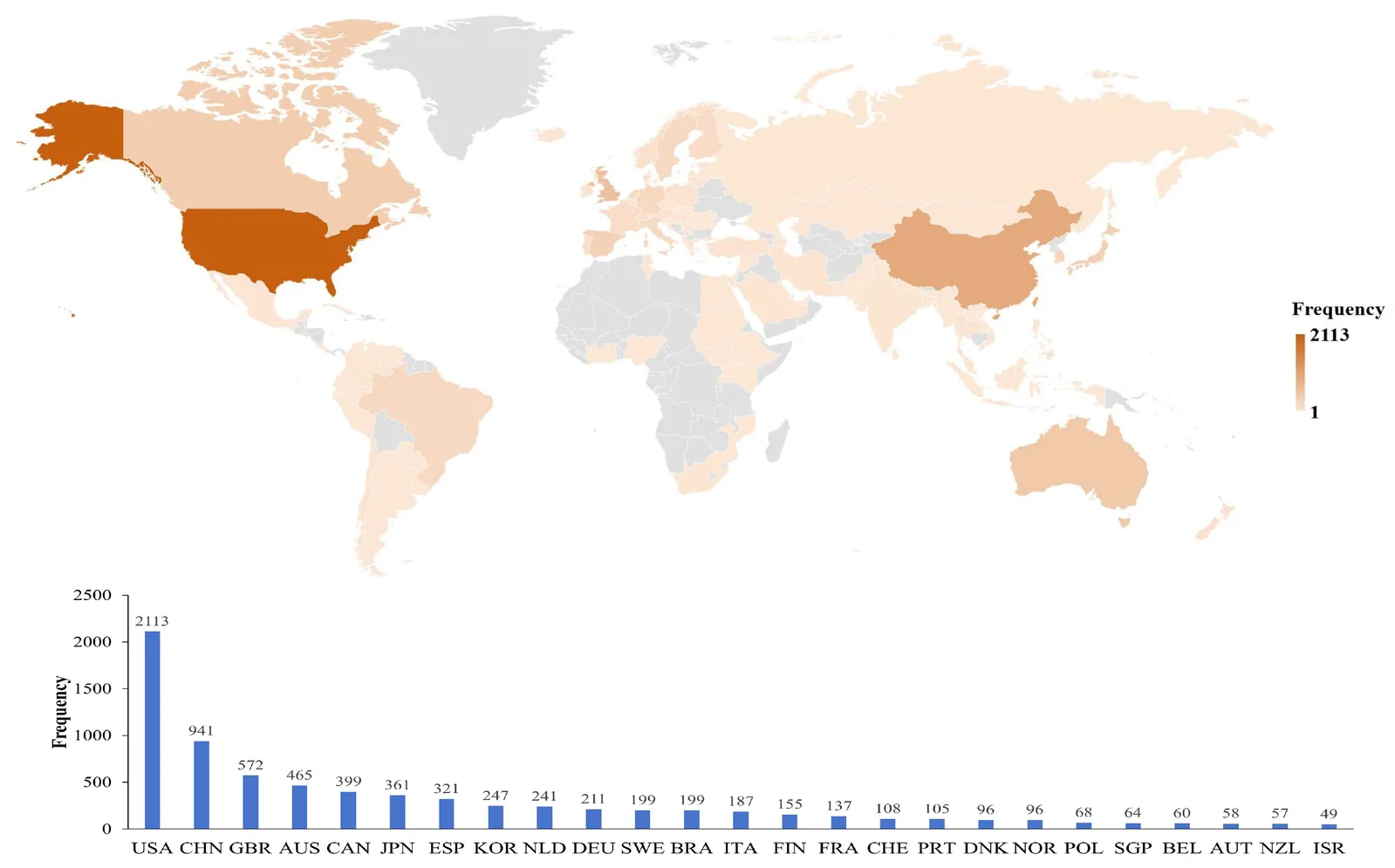
Distribution of the number of publications by country.

**Figure 4:**
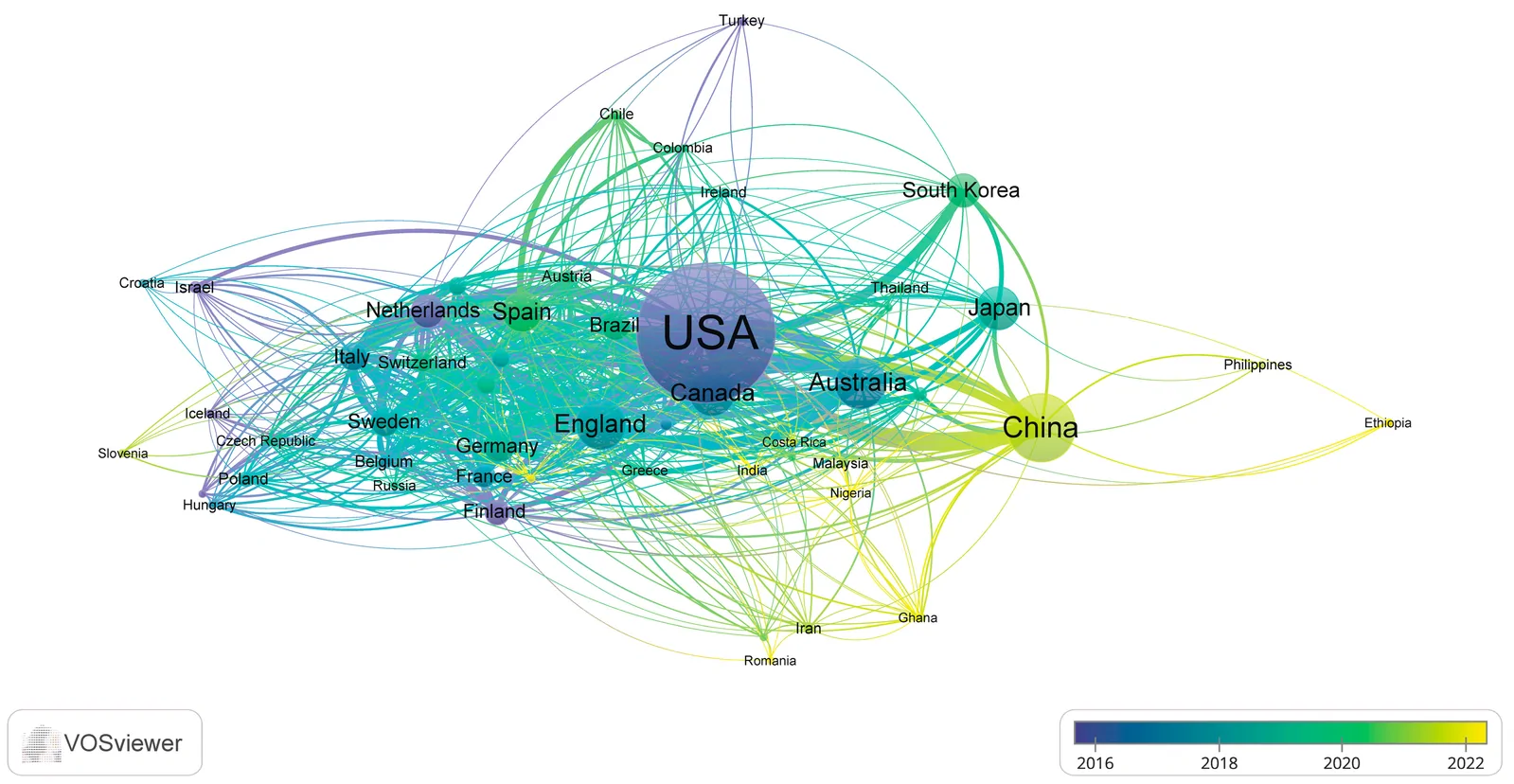
Countries appearing more than five times as shown by overlay visualization.

**Figure 5:**
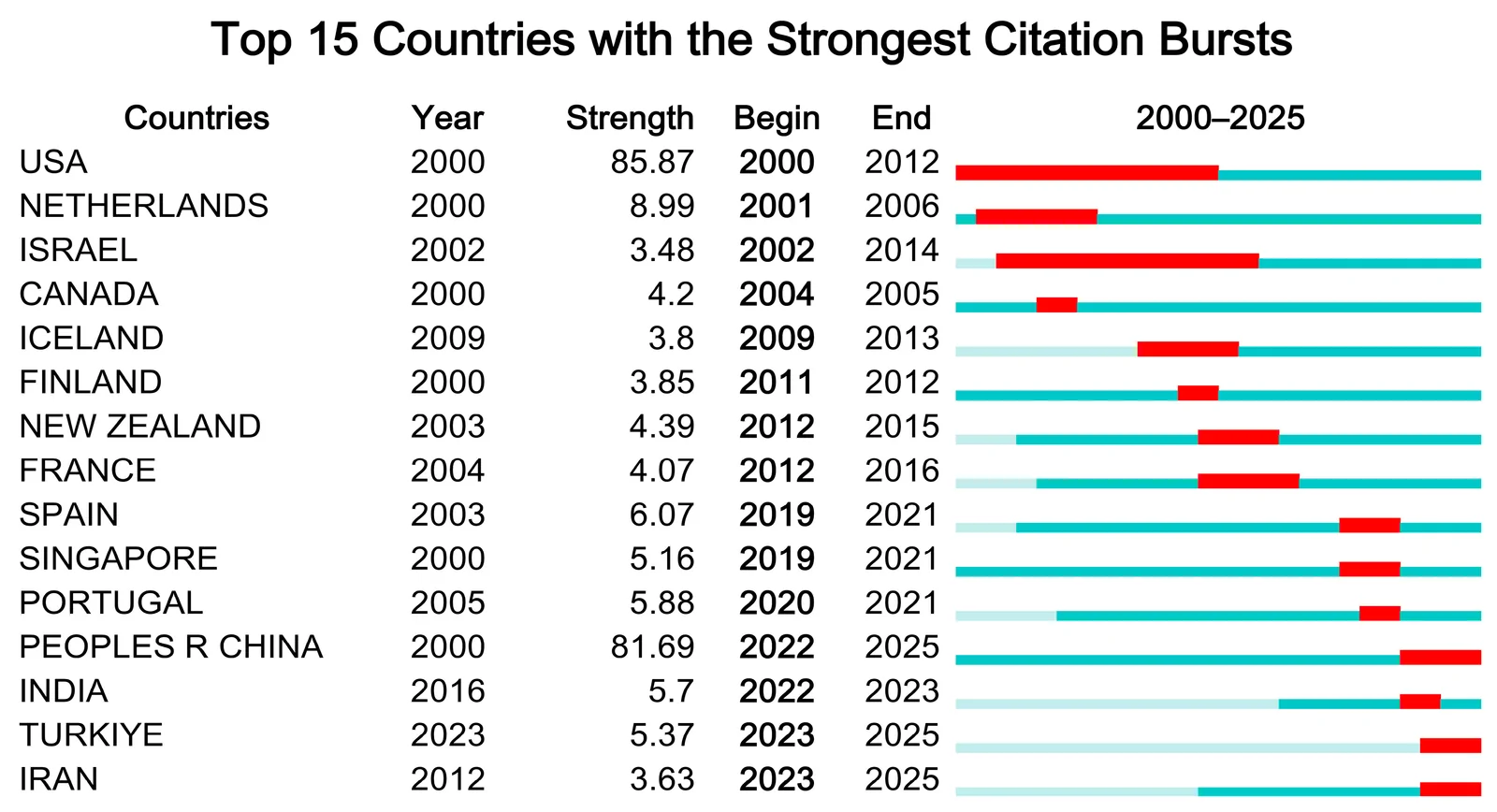
Countries with the strongest citation bursts.

**Figure 6:**
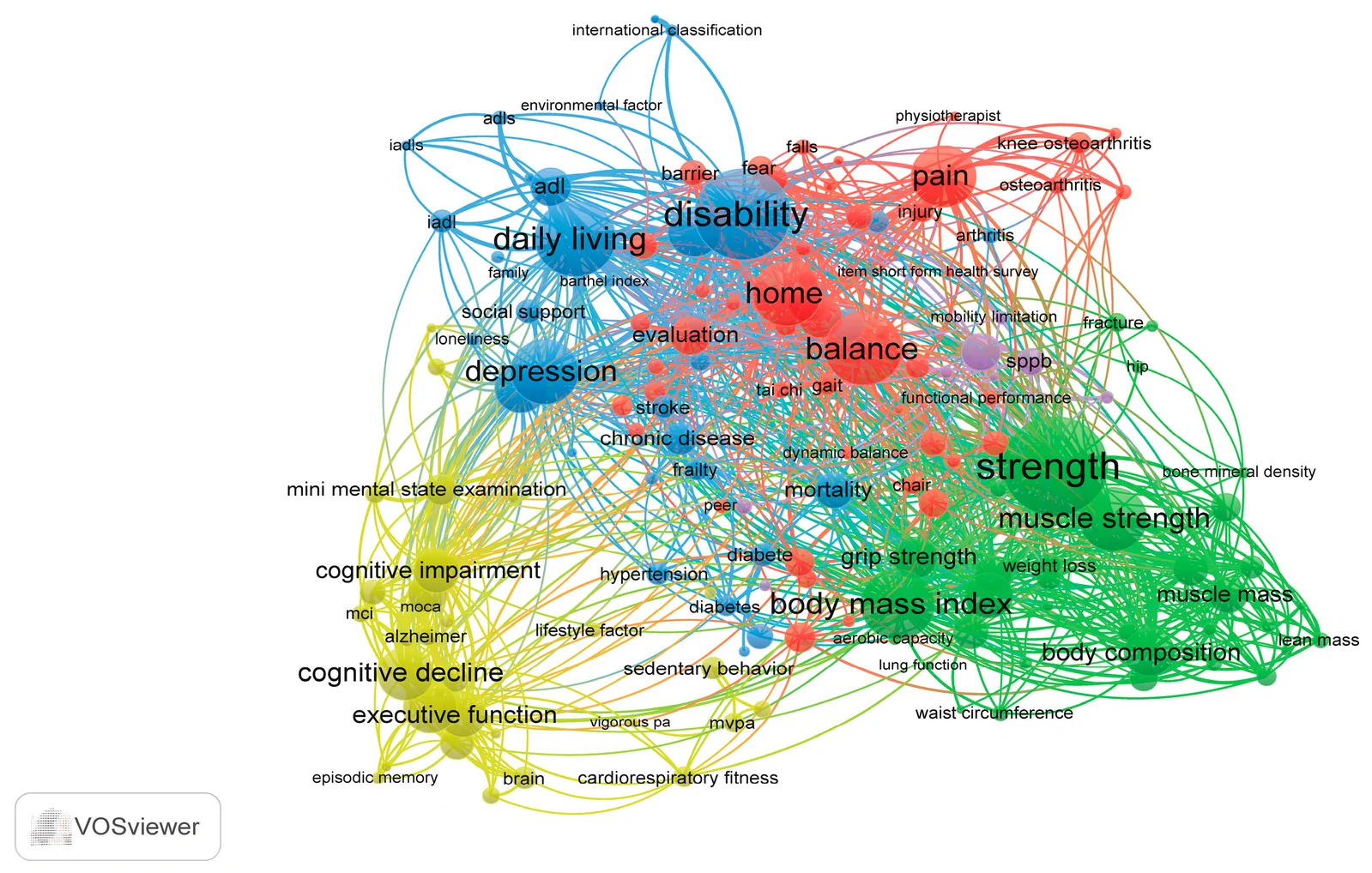
Co-occurrence terms in titles and abstracts as shown by network visualization. Different colors represent different clusters. Red is Cluster 1, green is Cluster 2, blue is Cluster 3, yellow is Cluster 4, and purple is Cluster 5.

**Figure 7:**
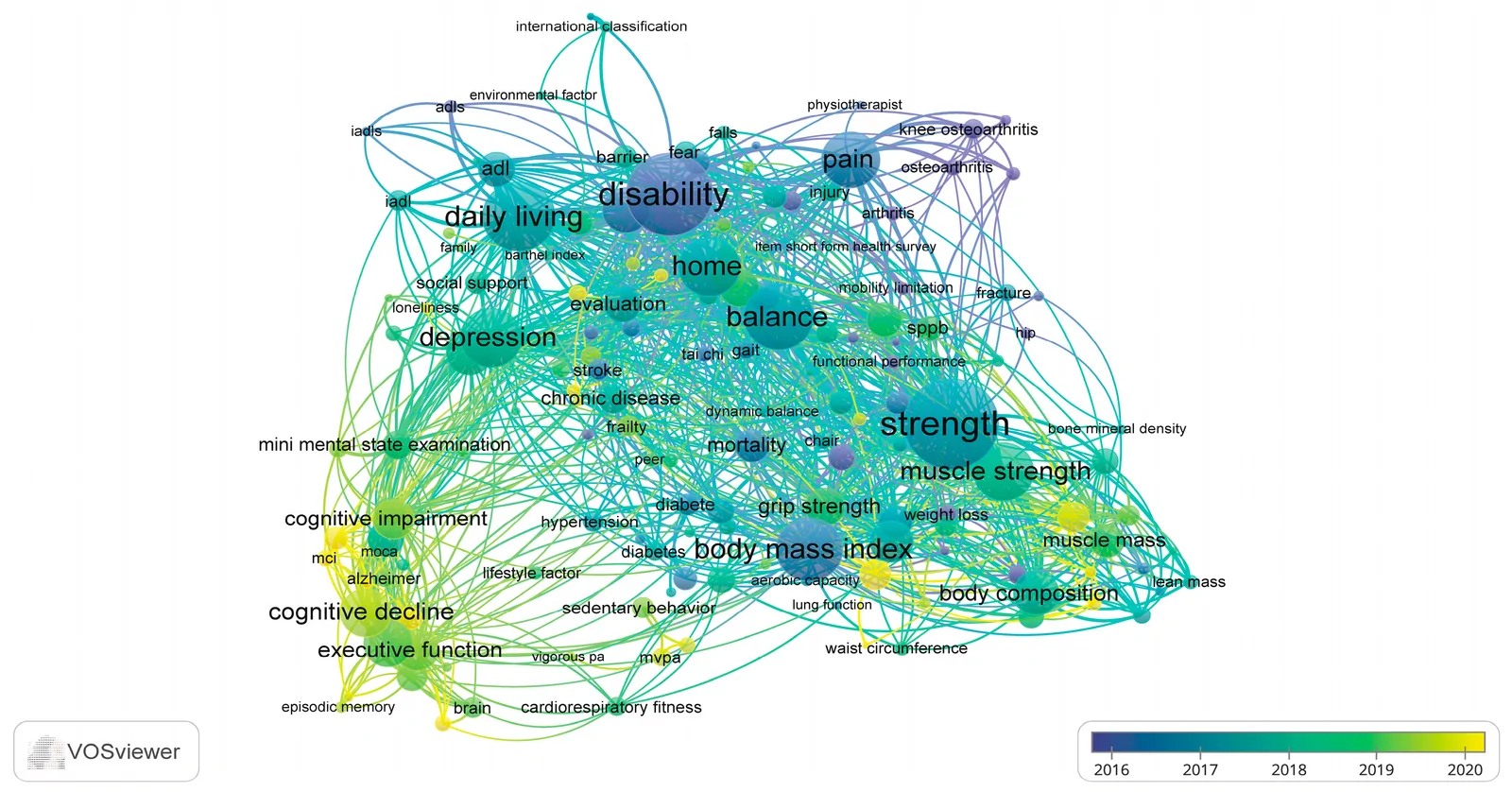
Co-occurrence terms in titles and abstracts as shown by overlay visualization.

**Figure 8:**
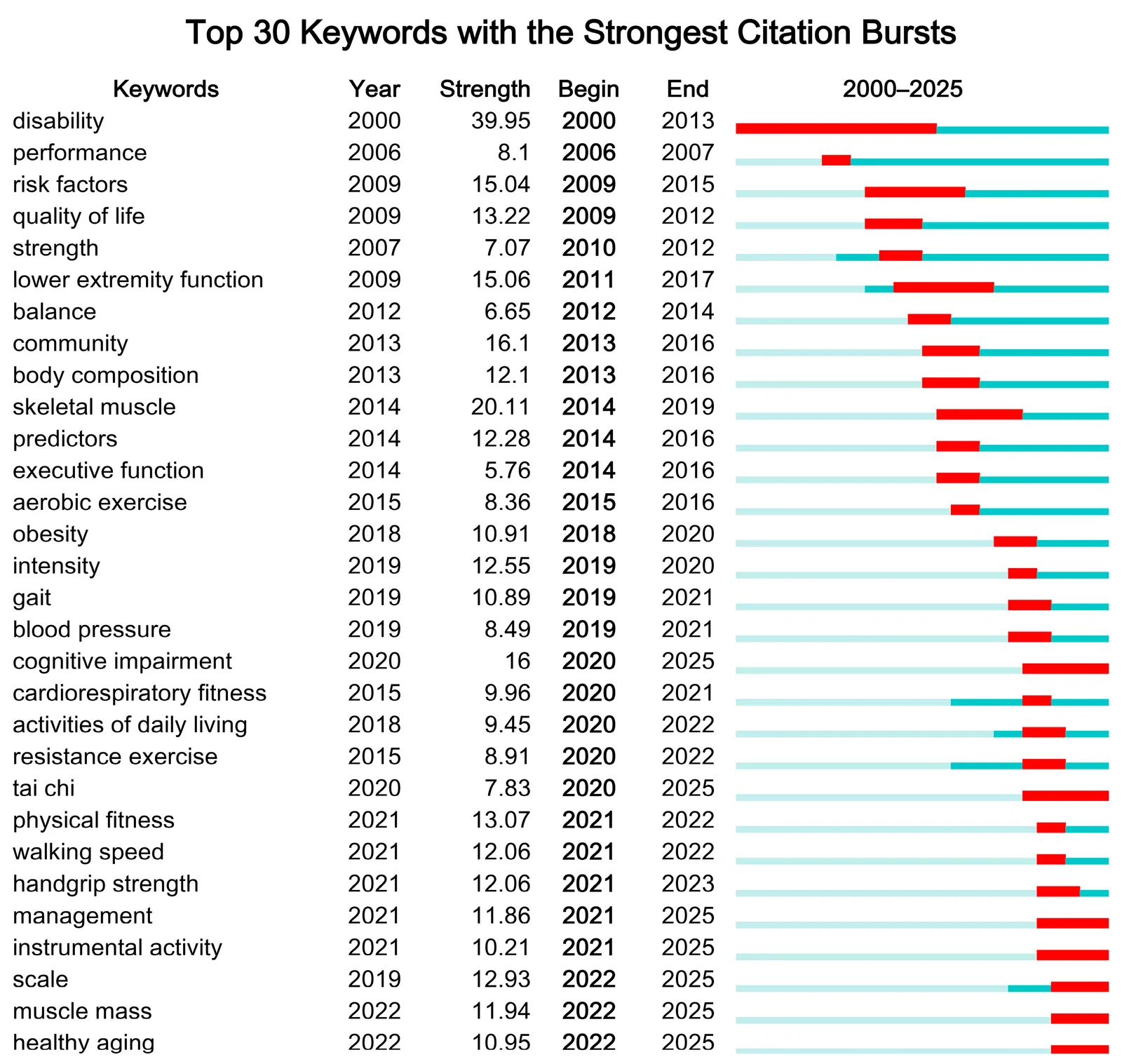
Keywords with the strongest citation bursts.

## Data Availability

No new data were created or analyzed in this study. Data sharing is not applicable.

## Supplementary Materials

The following supporting information can be downloaded at https://www.mdpi.com/article/10.3390/healthcare14193236/s1, Figure S1: Authors with more than 10 publications as shown by network visualization; Figure S2: Countries appearing more than 5 times as shown by network visualization; Figure S3: Countries cited as shown by overlay visualization; Figure S4: Institutions appearing more than 10 times as shown by network visualization; Figure S5: Co-occurrence for author keywords appearing more than 50 times as shown by network visualization; Figure S6: Co-occurrence for author keywords appearing more than 50 times as shown by overlay visualization; Figure S7: Co-occurrence for all keywords appearing more than 50 times as shown by network visualization; Figure S8: Co-occurrence for all keywords appearing more than 50 times as shown by overlay visualization; Table S1: Authors in the top 20 by number of publications; Table S2: Countries in the top 20 by number of publications; Table S3: Institutions in the top 20 by number of publications; Table S4: Top 20 most-cited sources; Table S5: Top 20 sources with the most-cited articles; Table S6: The 10 sources with the most cited and the most cited articles; Table S7: The 50 most-cited documents; Table S8: Results of the co-occurrence analysis for author keywords; Table S9: Results of the co-occurrence analysis for all keywords; Table S10: Results of co-occurrence analysis for terms in titles and abstracts.
