# Supplementary material for: Research Trends in Exercise Interventions for Functional Health in Older Adults: A Bibliometric Analysis (2000–2025)
Source: Healthcare (Basel). 2026 Oct 1;14(19):3236. doi: 10.3390/healthcare14193236 (PMC13665436; doi:10.3390/healthcare14193236)
Supplement: Supplementary file 1 [file healthcare-14-03236-s001.zip › Table S1-S10.pdf]

Table S1. Authors in the top 20 by number of publications.

Author	Links	Total link strength	Documents	Citations	Norm. Citations	Avg. pub. Year	Avg. Citations	Avg. norm. Citations	Centrality	Ranking
Newman, Anne B.	47	287	71	9786	126.52	2013.00	137.83	1.78	0.12	1
Kritchevsky, Stephen B.	47	268	57	7818	107.03	2012.54	137.16	1.88	0.04	2
Pahor, Marco	46	286	57	7348	90.80	2012.35	128.91	1.59	0.06	3
Rejeski, W. Jack	29	195	55	5289	77.83	2011.49	96.16	1.42	0.07	4
Simonsick, Eleanor M.	33	176	51	6967	78.95	2013.10	136.61	1.55	0.03	5
Guralnik, Jack M.	36	154	47	6536	86.47	2010.13	139.06	1.84	0.00	6
King, Abby C.	47	216	46	4793	80.87	2013.59	104.20	1.76	0.03	7
Rantanen, Taina	9	86	41	1256	32.75	2017.15	30.63	0.80	0.01	8
Harris, Tamara B.	27	131	39	7555	65.29	2009.10	193.72	1.67	0.00	9
Liu-ambrose, Teresa	23	87	37	1155	38.87	2018.24	31.22	1.05	0.11	10
Fielding, Roger A.	35	163	35	2676	57.73	2015.71	76.46	1.65	0.06	11
Ferrucci, Luigi	19	69	33	2846	42.54	2013.61	86.24	1.29	0.00	12
Glynn, Nancy W.	38	177	33	1298	34.67	2016.24	39.33	1.05	0.04	13
Manini, Todd M.	43	167	33	3014	57.20	2016.12	91.33	1.73	0.00	14
Shimada, Hiroyuki	15	109	33	1443	42.49	2018.00	43.73	1.29	0.04	15
Mcauley, Edward	13	83	32	3294	51.66	2015.59	102.94	1.61	0.03	16
Miller, Michael E.	31	175	30	2907	45.86	2011.63	96.90	1.53	0.00	17
Marsh, Anthony P.	25	116	28	1241	23.71	2013.54	44.32	0.85	0.01	18
Kramer, Arthur F.	18	77	27	3144	49.60	2017.15	116.44	1.84	0.06	19
Portegijs, Erja	6	70	27	744	20.78	2018.96	27.56	0.77	0.01	20

Table S2. Countries in the top 20 by number of publications.

Country	Links	Total link strength	Documents	Citations	Norm. Citations	Avg. pub. Year	Avg. Citations	Avg. norm. Citations	Centrality	Ranking
USA	43	647	2113	113322	2420.64	2016.18	53.63	1.15	0.33	1
CHN	31	224	941	19160	1048.30	2019.90	20.36	1.11	0.12	2
GBR	44	317	572	29324	826.70	2017.96	51.27	1.45	0.15	3
AUS	37	260	465	21099	573.53	2017.45	45.37	1.23	0.11	4
CAN	42	211	399	18404	516.26	2017.62	46.13	1.29	0.13	5
JPN	29	90	361	8973	338.83	2018.73	24.86	0.94	0.10	6
ESP	42	173	321	11403	441.78	2020.06	35.52	1.38	0.18	7
KOR	20	72	247	5998	243.92	2019.76	24.28	0.99	0.04	8
NLD	34	147	241	14895	285.33	2016.12	61.81	1.18	0.03	9
DEU	38	129	211	10756	333.48	2018.86	50.98	1.58	0.09	10
SWE	36	128	199	10908	293.64	2018.22	54.81	1.48	0.04	11
BRA	28	105	199	7246	260.67	2019.45	36.41	1.31	0.06	12
ITA	38	129	187	10524	293.50	2017.52	56.28	1.57	0.05	13
FIN	32	82	155	7463	173.82	2015.93	48.15	1.12	0.02	14
FRA	34	100	137	6866	220.01	2017.66	50.12	1.61	0.08	15
CHE	33	93	108	5166	163.79	2019.51	47.83	1.52	0.01	16
PRT	27	59	105	2892	113.93	2019.30	27.54	1.09	0.08	17
DNK	29	62	96	3511	109.89	2018.44	36.57	1.14	0.04	18
NOR	26	60	96	2498	92.37	2018.65	26.02	0.96	0.00	19
POL	21	25	68	2454	51.31	2018.25	36.09	0.75	0.02	20

Table S3. Institutions in the top 20 by number of publications.

Institutions	Links	Total link strength	Documents	Citations	Norm. Citations	Avg. pub. Year	Avg. Citations	Avg. norm. Citations	Centrality	Ranking
University of Pittsburgh	183	927	230	18390	299.87	2015.45	79.96	1.30	0.28	1
Wake Forest University	115	518	147	15140	213.35	2012.87	102.99	1.45	0.03	2
University of Illinois	133	320	135	8491	166.69	2016.22	62.90	1.23	0.16	3
UC San Francisco	128	454	119	12907	196.25	2014.45	108.46	1.65	0.09	4
National Institute on Aging	107	409	117	14993	163.57	2010.78	128.15	1.40	0.02	5
UC San Diego	119	410	103	5326	142.34	2017.33	51.71	1.38	0.06	6
Stanford University	110	493	100	7083	141.67	2015.58	70.83	1.42	0.02	7
University of Florida	98	415	92	6316	132.76	2015.60	68.65	1.44	0.03	8
University of Jyväskylä	84	193	92	3070	84.92	2016.47	33.37	0.92	0.08	9
Karolinska Institute	104	257	88	4732	137.02	2019.14	53.77	1.56	0.14	10
University of British Columbia	98	239	88	4778	117.12	2017.28	54.30	1.33	0.08	11
University of Sydney	80	196	88	4947	104.36	2016.16	56.22	1.19	0.07	12
Duke University	132	251	87	5184	127.89	2015.79	59.59	1.47	0.11	13
Johns Hopkins University	106	238	87	4379	93.81	2016.57	50.33	1.08	0.02	14
University College London	102	174	86	6242	149.05	2017.16	72.58	1.73	0.08	15
University of Melbourne	115	304	84	3383	92.94	2018.83	40.27	1.11	0.14	16
University of Washington	108	253	84	7945	153.50	2015.63	94.58	1.83	0.03	17
Northwestern University	87	322	83	5190	101.58	2015.59	62.53	1.22	0.01	18
University of Michigan	87	166	81	3701	98.47	2017.41	45.69	1.22	0.06	19
Vrije University Amsterdam	103	219	71	5692	86.34	2014.80	80.17	1.22	0.07	20

Table S4. Top 20 most-cited sources (Ranking by number of citations).

Ranking	Sources	Citations	Documents
1	journals of gerontology series a-biological sciences and medical sciences	17164	214
2	journal of the american geriatrics society	16583	167
3	jama-journal of the american medical association	7943	16
4	bmc geriatrics	5391	240
5	plos one	3780	104
6	lancet	3694	5
7	journals of gerontology series b-psychological sciences and social sciences	3487	43
8	journal of nutrition health & aging	3483	67
9	journal of the american medical directors association	3331	68
10	age and ageing	2746	54
11	archives of internal medicine	2708	13
12	gerontologist	2577	39
13	experimental gerontology	2477	79
14	international journal of environmental research and public health	2329	142
15	gerontology	2327	49
16	new england journal of medicine	2302	3
17	archives of physical medicine and rehabilitation	2248	45
18	archives of neurology	2224	3
19	journal of aging and physical activity	2199	137
20	physical therapy	2164	34

Table S5. Top 20 sources with the most-cited articles (Ranking by number of documents).

Ranking	Sources	Documents	Citations
1	bmc geriatrics	240	5391
2	journals of gerontology series a-biological sciences and medical sciences	214	17164
3	journal of the american geriatrics society	167	16583
4	international journal of environmental research and public health	142	2329
5	journal of aging and physical activity	137	2199
6	plos one	104	3780
7	aging clinical and experimental research	93	1841
8	archives of gerontology and geriatrics	85	2143
9	experimental gerontology	79	2477
10	bmc public health	73	1486
11	frontiers in public health	72	536
12	frontiers in aging neuroscience	69	1943
13	journal of the american medical directors association	68	3331
14	journal of nutrition health & aging	67	3483
15	bmj open	66	492
16	geriatrics & gerontology international	62	2021
17	age and ageing	54	2746
18	clinical interventions in aging	52	1416
19	journal of aging and health	52	1159
20	scientific reports	50	1079

Table S6. The 10 sources with the most cited and the most cited articles.

Sources	Documents	Citations
bmc geriatrics	240	5391
journals of gerontology series a-biological sciences and medical sciences	214	17164
journal of the american geriatrics society	167	16583
international journal of environmental research and public health	142	2329
journal of aging and physical activity	137	2199
plos one	104	3780
experimental gerontology	79	2477
journal of the american medical directors association	68	3331
journal of nutrition health & aging	67	3483
age and ageing	54	2746

Table S7. The 50 most-cited documents.

Ranking	Reference	Title	Citations
1	Ngandu (2015)	A 2 year multidomain intervention of diet, exercise, cognitive training, and vascular risk monitoring versus control to prevent cognitive decline in at-risk elderly people (FINGER): a randomised controlled trial. Lancet, 385(9984):2255-2263.	2419
2	Colcombe (2006)	Aerobic exercise training increases brain volume in aging humans. Journals of gerontology series a-biological sciences and medical sciences, 61(11), 1166-1170	1495
3	Newman (2006a)	Strength, but not muscle mass, is associated with mortality in the health, aging and body composition study cohort. Journals of gerontology series a-biological sciences and medical sciences, 61(1), 72-77	1298
4	Lautenschlager (2008)	Effect of physical activity on cognitive function in older adults at risk for alzheimer disease - a randomized trial. Jama-journal of the american medical association, 300(9), 1027-1037	1211
5	Deutz (2014)	Protein intake and exercise for optimal muscle function with aging: recommendations from the espen expert group. Clinical nutrition, 33(6), 929-936	1139
6	Pahor (2014)	Effect of structured physical activity on prevention of major mobility disability in older adults the life study randomized clinical trial. Jama-journal of the american medical association, 311(23), 2387-2396	1114
7	Wu (2010)	Identification of late-onset hypogonadism in middle-aged and elderly men. New england journal of medicine, 363(2), 123-135	1049
8	Laurin (2001)	Physical activity and risk of cognitive impairment and dementia in elderly persons. Archives of neurology, 58(3), 498-504	1048
9	Visser (2002)	Relationship of interleukin-6 and tumor necrosis factor-α with muscle mass and muscle strength in elderly men and women: the health abc study. Journals of gerontology series a-biological sciences and medical sciences, 57(5), m326-m332	1033
10	Larson (2006)	Exercise is associated with reduced risk for incident dementia among persons 65 years of age and older. Annals of internal medicine, 144(2), 73-81	983
11	Studenski (2003)	Physical performance measures in the clinical setting. Journal of the american geriatrics society, 51(3), 314-322	963
12	Pitta (2005)	Characteristics of physical activities in daily life in chronic obstructive pulmonary disease. American journal of respiratory and critical care medicine, 171(9), 972-977	909
13	Baker (2010)	Effects of aerobic exercise on mild cognitive impairment a controlled trial. Archives of neurology, 67(1), 71-79	871
14	Weuve (2004)	Physical activity, including walking, and cognitive function in older women. Jama-journal of the american medical association, 292(12), 1454-1461	826
15	Villareal (2011)	Weight loss, exercise, or both and physical function in obese older adults. New england journal of medicine, 364(13), 1218-1229	793

16	Fragala (2019)	Resistance training for older adults: position statement from the national strength and conditioning association. Journal of strength and conditioning research, 33(8), 2019-2052	779
17	Messier (2004)	Exercise and dietary weight loss in overweight and obese older adults with knee osteoarthritis - the arthritis, diet, and activity promotion trial. Arthritis and rheumatism, 50(5), 1501-1510	769
18	Newman (2006b)	Association of long-distance corridor walk performance with mortality, cardiovascular disease, mobility limitation, and disability. Jama-journal of the american medical association, 295(17), 2018-2026	761
19	Chekroud (2018)	Association between physical exercise and mental health in 1.2 million individuals in the usa between 2011 and 2015: a cross-sectional study. Lancet psychiatry, 5(9), 739-746	746
20	Rikli (2013)	Development and validation of criterion-referenced clinically relevant fitness standards for maintaining physical independence in later years. Gerontologist, 53(2), 255-267	742
21	Diamond (2016)	Conclusions about interventions, programs, and approaches for improving executive functions that appear justified and those that, despite much hype, do not. Developmental cognitive neuroscience, 18, 34-48	733
22	Friedman (2002)	Falls and fear of falling: which comes first? a longitudinal prediction model suggests strategies for primary and secondary prevention. Journal of the american geriatrics society, 50(8), 1329-1335	732
23	Izquierdo (2021)	International exercise recommendations in older adults (icfsr): expert consensus guidelines. journal of nutrition health & aging, 25(7), 824-853	716
24	Colcombe (2003)	Aerobic fitness reduces brain tissue loss in aging humans. Journals of gerontology series a-biological sciences and medical sciences, 58(2), 176-180	693
25	Messier (2013)	Effects of intensive diet and exercise on knee joint loads, inflammation, and clinical outcomes among overweight and obese adults with knee osteoarthritis the idea randomized clinical trial. Jama-journal of the american medical association, 310(12), 1263-1273	644
26	Cumming (2000)	Prospective study of the impact of fear of falling on activities of daily living, sf-36 scores, and nursing home admission. Journals of gerontology series a-biological sciences and medical sciences, 55(5), m299-m305	634
27	Davis (2011)	Exercise improves executive function and achievement and alters brain activation in overweight children: a randomized, controlled trial. Health psychology, 30(1), 91-98	634
28	Zeng (2017)	Survival, disabilities in activities of daily living, and physical and cognitive functioning among the oldest-old in china: a cohort study. Lancet, 389(10079), 1619-1629	622
29	Steffen (2008)	Test-retest reliability and minimal detectable change on balance and ambulation tests, the 36-item short-form health survey, and the unified parkinson disease rating scale in people with parkinsonism. Physical therapy, 88(6), 733-746	621
30	Yaffe (2001)	A prospective study of physical activity and cognitive decline in elderly women - women who walk. Archives of internal medicine, 161(14), 1703-1708	612
31	Lourida (2019)	Association of lifestyle and genetic risk with incidence of dementia. Jama-journal of the american medical association, 322(5), 430-437	593

32	Sepulveda-loyola (2020)	Impact of social isolation due to covid-19 on health in older people: mental and physical effects and recommendations. Journal of nutrition health & aging, 24(9):938-947	582
33	Menec (2003)	The relation between everyday activities and successful aging: a 6-year longitudinal study. Journals of gerontology series b-psychological sciences and social sciences, 58(2), s74-s82	568
34	Newman (2003)	Sarcopenia: alternative definitions and associations with lower extremity function. Journal of the american geriatrics society, 51(11), 1602-1609	566
35	Ensrud (2007)	Frailty and risk of falls, fracture, and mortality in older women: the study of osteoporotic fractures. Journals of gerontology series a-biological sciences and medical sciences, 62(7), 744-751	559
36	Haug (2015)	Smartphone use and smartphone addiction among young people in Switzerland. Journal of behavioral addictions, 4(4), 299-307	549
37	Montero-odasso (2005)	gait velocity as a single predictor of adverse events in healthy seniors aged 75 years and older. Journals of gerontology series a-biological sciences and medical sciences, 60(10), 1304-1309	544
38	Wolf (2005)	Health literacy and functional health status among older adults. Archives of internal medicine, 165(17), 1946-1952	540
39	Teri (2003)	Exercise plus behavioral management in patients with alzheimer disease - a randomized controlled trial. Jama-journal of the american medical association, 290(15), 2015-2022	536
40	Buchman (2012)	Total daily physical activity and the risk of ad and cognitive decline in older adults. Neurology, 78(17), 1323-1329	531
41	Cassilhas (2007)	The impact of resistance exercise on the cognitive function of the elderly. Medicine and science in sports and exercise, 39(8), 1401-1407	515
42	Abbott (2004)	Walking and dementia in physically capable elderly men. Jama-journal of the american medical association, 292(12), 1447-1453	514
43	Galvao (2010)	Combined resistance and aerobic exercise program reverses muscle loss in men undergoing androgen suppression therapy for prostate cancer without bone metastases: a randomized controlled trial. Journal of clinical oncology, 28(2), 340-347	513
44	Marottoli (2000)	Consequences of driving cessation: decreased out-of-home activity levels. Journals of gerontology series b-psychological sciences and social sciences, 55(6), s334-s340	508
45	Ferrucci (2002)	Change in muscle strength explains accelerated decline of physical function in older women with high interleukin-6 serum levels. Journal of the american geriatrics society, 50(12), 1947-1954	495
46	Satake (2016)	Validity of the kihon checklist for assessing frailty status. Geriatrics & gerontology international, 16(6), 709-715	488
47	Podewils (2005)	Physical activity, apoe genotype, and dementia risk: findings from the cardiovascular health cognition study. American journal of epidemiology, 161(7), 639-651	472
48	Bean (2002)	The relationship between leg power and physical performance in mobility-limited older people. Journal of the american geriatrics society, 50(3), 461-467	462

49	Villareal (2017)	Aerobic or resistance exercise, or both, in dieting obese older adults. new england journal of medicine, 376(20), 1943-1955	460
50	Hardy (2007)	Improvement in usual gait speed predicts better survival in older adults. journal of the american geriatrics society, 55(11), 1727-1734	459

Table S8. Results of the co-occurrence analysis for author keywords.

Keywords	Cluster	Links	Total link strength	Occurrences	Avg. pub. year	Avg. citations	Avg. norm. citations
cognition	1	34	231	327	2018.89	33.54	1.09
cognitive function	1	32	195	296	2020.55	19.50	0.96
depression	1	29	167	175	2019.51	24.27	1.00
dementia	1	25	154	155	2019.05	34.66	1.17
activities of daily living	1	29	123	144	2016.89	37.25	0.89
executive function	1	24	77	96	2018.42	38.90	0.99
mental health	1	22	72	96	2019.32	34.81	1.28
cognitive impairment	1	26	73	86	2020.40	18.94	1.07
mild cognitive impairment	1	21	70	73	2020.16	32.03	1.36
sedentary behavior	1	25	48	67	2020.51	22.36	0.92
cognitive decline	1	15	45	60	2021.35	22.25	1.01
alzheimer's disease	1	16	79	59	2019.07	38.49	1.18
quality of life	2	33	198	273	2017.70	31.30	0.83
rehabilitation	2	26	78	142	2015.33	37.69	0.68
falls	2	29	108	124	2018.60	39.13	1.31
balance	2	28	128	103	2017.10	31.95	0.65
walking	2	27	76	83	2017.18	32.69	0.79
osteoarthritis	2	15	47	64	2015.23	36.03	0.81
strength	2	19	58	60	2016.52	50.88	0.96
gait	2	25	65	59	2017.85	30.75	0.85
tai chi	2	20	51	57	2016.74	33.93	0.83
sarcopenia	3	29	169	197	2019.83	43.82	1.60
muscle strength	3	24	116	131	2017.94	31.89	1.04
obesity	3	27	107	119	2017.26	38.09	0.96
physical fitness	3	31	89	101	2017.12	32.09	0.82
resistance training	3	29	100	97	2018.15	32.03	1.12

body composition	3	20	67	85	2017.88	31.07	0.82
health-related quality of life	3	22	37	68	2017.09	31.96	0.90
nutrition	3	17	41	53	2018.66	49.79	1.40
frailty	4	32	176	254	2019.62	29.17	1.02
disability	4	29	133	171	2014.57	50.91	0.93
mobility	4	33	135	142	2016.72	38.45	0.91
physical performance	4	28	115	122	2017.71	31.57	0.96
healthy aging	4	18	42	73	2021.05	22.05	1.15
mortality	4	23	55	62	2015.69	58.97	1.12
gait speed	4	20	49	59	2018.34	45.90	1.21

Table S9. Results of the co-occurrence analysis for all keywords (top50).

Keywords	Cluster	Links	Total link strength	Occurrences	Avg. pub. year	Avg. citations	Avg. norm. citations
dementia	1	83	1760	608	2019.04	36.30	1.23
cognitive function	1	86	1353	487	2019.82	25.98	1.02
impairment	1	86	1202	392	2019.10	42.28	1.20
cognition	1	85	1088	359	2019.04	33.07	1.08
fitness	1	90	904	324	2016.47	53.39	1.03
alzheimers-disease	1	79	939	300	2018.34	54.94	1.24
aerobic exercise	1	87	672	208	2017.47	53.31	1.34
executive function	1	73	549	172	2018.65	33.94	0.94
cognitive impairment	1	78	518	167	2019.35	32.98	1.06
memory	1	69	488	163	2018.73	36.89	1.08
sedentary behavior	1	81	402	163	2020.50	21.90	0.92
brain	1	72	462	159	2019.25	31.01	0.99
mild cognitive impairment	1	68	404	130	2019.55	34.27	1.37
cardiorespiratory fitness	1	65	340	121	2017.48	38.88	0.90
cognitive decline	1	60	313	107	2019.50	32.83	1.00
cardiovascular-disease	1	66	271	105	2016.38	62.12	1.08
falls	2	84	946	349	2017.66	36.21	0.96
balance	2	80	942	322	2017.29	30.63	0.77
rehabilitation	2	84	636	244	2016.15	35.75	0.73
walking	2	83	600	224	2016.94	41.64	0.92
gait	2	70	435	140	2018.25	27.21	0.82
osteoarthritis	2	61	344	120	2015.43	31.27	0.69
pain	2	71	334	115	2016.87	43.31	0.90
self-efficacy	2	68	295	107	2016.13	37.13	0.86
knee osteoarthritis	2	63	316	106	2014.94	57.39	1.18
adherence	2	74	262	105	2019.09	17.85	0.93

strength	3	88	1393	491	2017.30	38.72	0.98
sarcopenia	3	79	953	326	2019.67	38.33	1.40
muscle strength	3	88	993	324	2017.27	32.77	0.93
obesity	3	80	646	225	2017.21	38.80	0.96
body-composition	3	72	569	184	2016.27	50.79	1.00
skeletal-muscle	3	63	475	162	2016.61	57.52	1.36
resistance exercise	3	73	393	128	2017.55	49.98	1.54
nutrition	3	68	293	104	2018.80	38.96	1.13
muscle mass	3	55	328	103	2018.71	44.04	1.40
quality-of-life	4	89	1344	525	2017.30	38.62	1.00
depression	4	85	1131	401	2018.84	29.68	1.01
quality of life	4	86	726	273	2017.70	31.30	0.83
community	4	75	616	227	2015.82	45.93	0.83
depressive symptoms	4	75	367	139	2018.41	42.14	1.44
symptoms	4	74	326	112	2018.22	22.76	0.98
disability	5	90	2270	847	2016.12	42.88	0.92
mortality	5	89	1534	629	2016.95	46.04	1.10
mobility	5	86	1260	408	2017.15	38.64	0.96
frailty	5	83	977	392	2019.42	30.71	1.03
lower-extremity function	5	78	830	278	2015.72	49.35	1.01
gait speed	5	83	678	200	2018.36	41.84	1.10
activities of daily living	5	73	428	144	2016.89	37.25	0.89
grip strength	5	76	512	143	2019.27	28.74	1.01
walking speed	5	70	360	106	2018.04	32.03	0.84

Table S10. Results of co-occurrence analysis for terms in titles and abstracts (top50).

Terms	Cluster	Links	Total link strength	Occurrences	Avg. pub. year	Avg. citations	Avg. norm. citations
balance	1	158	2972	562	2017.19	33.42	0.82
home	1	157	2441	497	2017.41	40.36	1.00
pain	1	152	2245	463	2016.67	42.69	0.95
adherence	1	153	1524	306	2018.68	29.58	0.98
evaluation	1	155	1276	279	2017.55	35.60	0.76
feasibility	1	153	1401	263	2019.19	21.56	0.81
physical fitness	1	145	987	202	2018.08	24.02	0.73
endurance	1	138	988	190	2016.06	45.10	1.03
flexibility	1	148	1086	190	2017.33	31.51	0.94
environment	1	135	685	187	2018.47	29.74	0.91
self efficacy	1	135	836	187	2016.62	32.46	0.81
rehabilitation	1	143	756	173	2017.72	37.27	0.87
distance	1	136	716	169	2016.65	42.73	0.99
functional capacity	1	150	904	167	2017.54	45.96	1.21
barrier	1	131	688	166	2017.93	31.96	0.95
fear	1	132	813	161	2017.52	45.06	1.03
strength	2	159	4421	798	2017.12	46.27	1.12
body mass index	2	159	2734	538	2016.62	45.41	1.00
muscle strength	2	158	2929	488	2018.09	40.57	1.11
body composition	2	148	2074	350	2017.90	49.40	1.11
bmi	2	157	1937	340	2017.55	35.62	0.95
grip strength	2	158	1638	303	2018.66	38.42	1.01
muscle mass	2	147	1640	256	2018.79	55.59	1.53
obesity	2	153	1544	249	2017.15	52.50	1.46
handgrip strength	2	153	1385	236	2020.11	21.03	0.99
sarcopenia	2	143	1504	233	2019.76	52.68	1.76

muscle	2	133	990	192	2018.04	32.94	1.09
protein	2	141	1060	189	2018.37	55.41	1.45
disability	3	158	3735	725	2016.32	49.56	1.14
daily living	3	157	3244	597	2017.39	38.89	1.03
depression	3	158	2522	496	2017.93	43.72	1.29
limitation	3	158	1824	368	2016.54	50.02	1.08
depressive symptom	3	158	1712	359	2018.14	34.08	1.05
adl	3	147	1625	277	2017.26	32.25	0.99
mortality	3	149	1248	258	2016.67	68.83	1.48
comorbidity	3	150	1272	239	2016.82	49.15	1.09
chronic disease	3	152	1152	228	2017.82	39.61	1.35
cardiovascular disease	3	134	796	170	2016.77	58.81	1.52
cognitive decline	4	148	1693	397	2019.53	44.84	1.39
memory	4	145	1724	381	2018.97	42.68	1.39
executive function	4	142	1524	331	2019.24	43.05	1.34
cognitive impairment	4	147	1554	319	2019.31	51.03	1.37
cognitive functioning	4	128	1046	266	2017.93	50.73	1.31
cognitive performance	4	136	984	230	2019.13	38.93	1.10
cognitive	4	140	1109	228	2018.79	36.07	1.08
mini mental state examination	4	145	1271	218	2018.52	42.11	1.27
mild cognitive impairment	4	127	937	168	2020.29	39.37	1.41
cognitive health	4	123	701	167	2020.67	18.46	1.12
short physical performance battery	5	150	1561	269	2018.68	37.68	1.09
sppb	5	146	1166	189	2018.69	31.93	1.01
